# Validity of activity wristbands for estimating daily physical activity in primary schoolchildren under free-living conditions: School-Fit study

**DOI:** 10.3389/fpubh.2023.1211237

**Published:** 2023-07-24

**Authors:** Daniel Mayorga-Vega, Carolina Casado-Robles, Santiago Guijarro-Romero, Jesús Viciana

**Affiliations:** ^1^Departamento de Didáctica de las Lenguas, las Artes y el Deporte, Facultad de Ciencias de la Educación, Universidad de Málaga, Málaga, Spain; ^2^Department of Physical Education and Sport, University of Granada, Granada, Spain; ^3^Department of Didactic of Musical, Plastic and Corporal Expression, University of Valladolid, Valladolid, Spain

**Keywords:** consumer-wearable activity trackers, wrist-worn wearables, fitness trackers, agreement, steps, moderate-to-vigorous physical activity, physical activity recommendations, children

## Abstract

**Introduction:**

The use of activity wristbands to monitor and promote schoolchildren's physical activity (PA) is increasingly widespread. However, their validity has not been sufficiently studied, especially among primary schoolchildren. Consequently, the main purpose was to examine the validity of the daily steps and moderate-to-vigorous PA (MVPA) scores estimated by the activity wristbands Fitbit Ace 2, Garmin Vivofit Jr 2, and the Xiaomi Mi Band 5 in primary schoolchildren under free-living conditions.

**Materials and methods:**

An initial sample of 67 schoolchildren (final sample = 62; 50% females), aged 9–12 years old (mean = 10.4 ± 1.0 years), participated in the present study. Each participant wore three activity wristbands (Fitbit Ace 2, Garmin Vivofit Jr 2, and Xiaomi Mi Band 5) on his/her non-dominant wrist and a research-grade accelerometer (ActiGraph wGT3X-BT) on his/her hip as the reference standard (number of steps and time in MVPA) during the waking time of one day.

**Results:**

Results showed that the validity of the daily step scores estimated by the Garmin Vivofit Jr 2 and Xiaomi Mi Band 5 were good and acceptable (e.g., MAPE = 9.6/11.3%, and lower 95% IC of ICC = 0.87/0.73), respectively, as well as correctly classified schoolchildren as meeting or not meeting the daily 10,000/12,000-step-based recommendations, obtaining excellent/good and good/acceptable results (e.g., Garmin Vivofit Jr 2*, k* = 0.75/0.62; Xiaomi Mi Band 5, *k* = 0.73/0.53), respectively. However, the Fitbit Ace 2 did not show an acceptable validity (e.g., daily steps: MAPE = 21.1%, and lower 95% IC of ICC = 0.00; step-based recommendations: *k* = 0.48/0.36). None of the three activity wristbands showed an adequate validity for estimating daily MVPA (e.g., MAPE = 36.6–90.3%, and lower 95% IC of ICC = 0.00–0.41) and the validity for the MVPA-based recommendation tended to be considerably lower (e.g., *k* = −0.03–0.54).

**Conclusions:**

The activity wristband Garmin Vivofit Jr 2 obtained the best validity for monitoring primary schoolchildren's daily steps, offering a feasible alternative to the research-grade accelerometers. Furthermore, this activity wristband could be used during PA promotion programs to provide accurate feedback to primary schoolchildren to ensure their accomplishment with the PA recommendations.

## 1. Introduction

Engaging in habitual physical activity (PA) is largely recognized as a key indicator of health among school-aged children (1). For instance, there is strong evidence that, among schoolchildren, habitual moderate-to-vigorous PA (MVPA) levels are favorably associated with several health markers, such as cardiorespiratory and musculoskeletal fitness, cardiometabolic health, adiposity, motor skill development, bone health, cognitive function, academic outcomes, and depression (1). Moreover, in the last years, there is evidence that total PA is also favourably related to several health outcomes among schoolchildren (2), representing steps per day a common and credible output (3, 4).

The World Health Organization (1) recommends that schoolchildren should do at least an average of 60 min per day of MVPA, mainly involving a variety of aerobic activities, such as brisk walking or running. However, since these public health guidelines are not easily understood by both schoolchildren and their parents (5), the MVPA-based guidelines have also been translated to a simple recommendation among schoolchildren of achieving at least about 10,000 (6) or 12,000 steps per day (7). Unfortunately, worldwide under 20% of schoolchildren meet the PA recommendations (8). Moreover, since behaviors established during childhood are likely to track into adulthood, unhealthy PA habits (i.e., not meeting the above-mentioned PA guidelines) during this period might also negatively influence adult health status (9). In line with this scenario, a current global action plan in public health is to reduce by 15% the number of children who are physically inactive by 2030 (10).

Consumer-wearable activity trackers have emerged with the main purpose of monitoring and promoting users' habitual PA (11). Over the last decade, these devices have become very popular, with global wearable device sales reaching an estimated over 500 million worldwide (12). Consumer-wearable activity trackers are electronic devices worn on the body as an accessory monitoring and recording daily PA outputs such as step counts, distance or time in intensity-related PA, and providing users real-time behavioral feedback (11). Moreover, these devices often include other features that also may be facilitators of users' positive behavior change such as personalized goal-setting (based on daily steps or minutes of MVPA), self-efficacy, peer comparison, or social support (11). Thus, with the increasing proliferation of consumer-wearable activity trackers, together with the above-mentioned characteristics, stakeholders such as researchers, paediatrics, physical education teachers or parents are interested in leveraging consumer-wearable activity trackers as a means to monitor and promote healthy habits of PA in schoolchildren (11, 13). Among the different consumer-wearable activity trackers available (e.g., activity wristbands, smartwatches, pedometers, or smartphones), activity wristbands have shown to be one of the most valued and used type of these devices, especially by children (13, 14). Particularly, these devices are characterized because they include real-time feedback, an attractive display, low weight and price, and goal alerts, among others (15, 16). In this line, recently, Casado-Robles et al. (11) in a systematic review and meta-analysis found that the activity wristbands were the most effective kind of consumer-wearable activity trackers for promoting schoolchildren's daily steps and MVPA levels. Therefore, activity wristbands potentially represent a feasible instrument to objectively monitor and promote schoolchildren's daily PA (11, 17).

Before using a particular activity wristband for monitoring and/or promoting daily PA, its validity should be assessed and considered adequate in the target population (18, 19). Validity of the activity wristbands scores can be studied by examining the agreement between the scores from the index test (i.e., activity wristband) and those from the “reference standard” under three different testing conditions: controlled (also known as laboratory), structured free-living (also known as simulated free-living or semi-free living), and free-living (also known as unstructured free-living) (20). The free-living condition, which involves participants wearing the activity wristband during “normal” daily life, is especially important to be examined because it considers the ecological validation of these technologies (20). Nowadays video-based counting and oxygen uptake measured by a portable indirect calorimetry system are considered the “reference standard” for assessing steps and MVPA, respectively (20, 21). However, since these methods commonly are not feasible under free-living conditions (21), today research-grade accelerometers are considered as the most appropriate alternative (21–24).

Despite the use of activity wristbands being increasingly widespread, evidence of its validity is still limited and contradictory among primary schoolchildren. For instance, while the activity wristbands Fitbit Charge HR and Xiaomi Mi Band showed adequate-excellent validity for estimating steps (25, 26), the Fibit Flex 2 and Movband Model 2 ones did not show adequate results (27, 28). As regards the assessment of MVPA, all the previously studied activity wristbands (i.e., Fitbit Charge HR and Flex 2, and Xiaomi Mi Band) showed inadequate validity among primary schoolchildren (26, 27, 29). Despite the fact that activity wristbands could be not valid for estimating the exact values of PA levels (i.e., as a continuous variable), from a health promotion perspective, the main interest is knowing if activity wristbands are simply valid for classifying schoolchildren as meeting or not meeting the PA recommendations (i.e., as a dichotomous variable) (13). Furthermore, since different kinds of activity wristbands could be used in the same context due to economic constrains (e.g., monitoring or promoting PA in the physical education setting or large-scale research studies) (30, 31), the agreement between activity wristbands (i.e., comparability) should be also studied (14). Unfortunately, to our knowledge, there are no previous topic-related studies with primary schoolchildren. Furthermore, although currently there are activity wristbands specially designed for primary schoolchildren such as the Fitbit Ace and Garmin Vivofit Jr, no previous study on the validity with those activity wristbands was found.

Consequently, the main purpose of the present study was to examine the validity of the daily steps and MVPA scores estimated by the activity wristbands Fitbit Ace 2, Garmin Vivofit Jr 2, and Xiaomi Mi Band 5 using the ActiGraph accelerometers as the reference standard in primary schoolchildren under free-living conditions. The secondary purpose was to examine the comparability of the three above-mentioned activity wristbands for estimating day steps and MVPA in primary schoolchildren under free-living conditions.

## 2. Materials and methods

### 2.1. Participants

The present study is reported according to the GRRAS guidelines (19). The protocol of the present study conforms to the Declaration of Helsinki statements (64th WMA, Brazil, October 2013) and it was first approved by the Ethical Committee for Human Studies at the University of Granada (1252/CEIH/2020). Then, the principals and the physical education teachers of a public primary school chosen by convenience were contacted. They were informed about the project, and permission to conduct the study was requested. After the approval of the school was obtained, all the schoolchildren and their legal guardians were fully informed about the features of the project. Schoolchildren's verbal informed assents and their legal guardians' signed written informed consents were obtained before taking part in the study.

The present study followed a cross-sectional design. A total of 75 schoolchildren from 4^th^ to 6^th^ grade (i.e., 9–12 years old) enrolled in the selected school were invited to participate in the present study. The school was located in the town (i.e., urban area) of Motril (Granada, Spain). The following inclusion criteria were considered: (a) being enrolled in the 4^th^ to 6^th^ grade at the primary education level (i.e., target grades according to study aim); (b) being free of any health disorder that would make them unable to engage in PA normally; (c) providing the corresponding verbal informed assents of the schoolchildren, and (d) presenting the corresponding signed written informed consents of their legal guardians. The following exclusion criteria were considered: (a) not having completed and valid data from the three activity wristbands, and/or (b) not having completed and valid data from the accelerometer.

A priori sample size calculation was estimated with the Arifin's web-based sample size calculator (32). Based on steps values, parameters were set as follows: ICC, ρ_0_ = 0.70 (33); ρ_1_ = 0.85 (34), α = 0.05, 1 – β = 0.80, *k* = 2, dropout = 23% (35). Kappa, *k*_0_ = 0.40 (36); *k*_1_ = 0.80 (37), *p* = 0.25 (8), α = 0.05, 1 – β = 0.80, *k* = 2, dropout = 23% (35). A final sample size of at least 53 schoolchildren (minimum initial sample size = 69) was estimated. In addition to exceeding the minimum required sample size, the aim for each study sampling was to obtain a sample balanced by grade and gender.

### 2.2. Measures

#### 2.2.1. Demographic characteristics

Schoolchildren's grade (4^th^, 5^th^ or 6^th^), gender (males/females), age (in years) and non-dominant hand (left/right) information was self-reported in a written questionnaire.

#### 2.2.2. Anthropometric

Schoolchildren's body mass (kg) and height (cm) were first measured following the International Standards for Anthropometric Assessment (38). Schoolchildren's body mass and height were measured in shorts, T-shirts, and barefoot. For the body mass measure, the Schoolchildren stood in the centre of the scale (Seca, Ltd., Hamburg, Germany; accuracy = 0.1 kg) without support and with the weight distributed evenly on both feet. For the body height assessment, schoolchildren stood with their feet together with the heels, buttocks and upper part of the back touching the stadiometer (Holtain Ltd., Crymmych, Pembs, United Kingdom; accuracy = 0.1 cm), and with the head placed in the Frankfort plane. Each measurement was performed twice and the mean was recorded (38). Then, the body mass index was calculated as body mass divided by body height squared (kg/m^2^). Finally, schoolchildren's body weight status was categorized by gender- and age-adjusted body mass index thresholds as overweight/obesity or non-overweight/obesity (39). Body mass index and body weight status scores have shown high evidence supporting validity among schoolchildren (39).

#### 2.2.3. Activity wristbands

Participants' daily steps and MVPA levels were estimated by the activity wristbands Fitbit Ace 2 (Fitbit, San Francisco, SF, USA), Garmin Vivofit Jr 2 (Garmin, Kansas, KS, USA), and Xiaomi Mi Band 5 (Xiaomi, Pekin, China). Regarding the number of activity wristbands, it was considered that three devices was the maximum number of wristbands that did not interfere with schoolchildren's daily activities (i.e., PA prevalence and patterns) and their correct measurement (i.e., adequate wrist adjustment and natural arm swing). In this line, the total mass of the three activity wristbands was not high (37.5 grams). According to the user manual of each device brand, the activity wristbands were fit snugly on the top of participants' wrist of the non-dominant hand, close, and above the wrist bone (3.91 cm width). As regards the particular chosen activity wristbands, the criteria were to study: (a) the most worldwide used display-based activity wristbands brands (40) (IDC's Worldwide Quarterly Wearable Device Tracker reports from 2017 to 2020); (b) choosing devices models with affordable prices (based on launch prices in Spain; Fitbit Ace 2 ≈ 70€; Garmin Vivofit Jr 2 ≈ 70€; Xiaomi Mi Band 5 ≈ 35€); and (c) when they were available, models designed specifically for children (i.e., Garmin Vivofit Jr 2 and Fitbit Ace 2).

The three chosen devices are characterized to be small and light-weight activity wristbands (Fitbit Ace 2: 2.27 × 1.00 × 0.30 cm, 20.0 g; Garmin Vivofit Jr 2: 1.1 × 1.1 × 0.9 cm, 17.5 g; Xiaomi Mi Band 5: 4.69 × 1.81 × 1.24 cm, 11.9 g), based in tri-axial built-in accelerometers. Each activity wristband has its proprietary algorithmic to estimate the daily steps taken and the minutes engaged in MVPA. Apart from the possibility to record data immediately from the screen, they can also be synchronized via Bluetooth to their specific applications to download and store data. Regarding the data scoring, steps (number) were registered as directly stored in their specific applications. However, specific information regarding algorithms used to calculate the time (minutes) engaged in MVPA is not made publicly available by the companies. Therefore, in line with the assumption made by previous topic-related studies (14, 27, 41), in the present study MVPA scores (minutes) were calculated as follows: (a) Fitbit Ace 2 and Garmin Vivofit Jr 2, “minutes of activity” was used as MVPA, and (b) Xiaomi Mi Band 5, MVPA and “brisk walking” were calculated by adding up the total time spent on all the bouts of “moderate activity”/“vigorous activity” and “fast walking”, respectively [according to the Youth Compendium of PA (42), “brisk walking” corresponds to MVPA].

#### 2.2.4. Accelerometer

Participants' reference standards of daily steps and MVPA scores were determined by wGT3X-BT accelerometers (ActiGraph, LLC, Pensacola, FL, USA). The ActiGraph model wGT3X-BT is a small (4.6 × 3.3 × 1.5 cm), light-weight (19 g), tri-axial accelerometer. Accelerometers were adjusted on the schoolchildren's right hips. Initializing, downloading, wear time validation, and scoring were performed using the ActiLife Lifestyle Monitoring System Software version 6.13.3 (ActiGraph, LLC, Pensacola, FL, USA). Accelerometers were initialized with a sample ratio of 30 Hz (43, 44). Since schoolchildren's behavior patterns are characterized by short bursts of quickly changing activity, data download was carried out with 15-second *epochs* (24). Valid wear time was set as equal to or higher than 600 min per day (24), with non-wear periods set as 60 min or more of consecutive zero-count *epochs* with up to 2 min spike tolerance (45).

Regarding the data scoring, steps (number) were assessed by within-instrument processing of the number of cycles in the accelerometer signal or *cycle counts*. The time (minutes) engaged in MVPA was calculated as ≥2,296 *counts*/min (43). According to the cross-validation study performed by Trost et al. (44), this threshold has demonstrated the best evidence supporting score validity for assessing MVPA among schoolchildren. Moreover, Romanzini et al. (46) later provided more support for the continued use of the ≥ 2,296 *counts*/min threshold among schoolchildren. Finally, schoolchildren's steps and MVPA were dichotomized as meeting or not meeting the daily recommendation of at least 10,000/12,000 steps (6, 7) and 60 min of MVPA (1), respectively. ActiGraph accelerometer scores have shown high evidence supporting validity for assessing steps and MVPA among schoolchildren (22, 23, 46).

### 2.3. Procedure

Data collection was carried out by the same researcher, using the same instruments and protocols. Firstly, participants' demographic characteristics and anthropometric measurements were recorded. Then, activity wristbands and accelerometers were adjusted from Monday to Thursday, and data were downloaded and batteries charged on Fridays. Due to the limitations of material resources, waves of 5–6 schoolchildren per day were carried out. For each wave, schoolchildren were met at 8:40 a.m. in the assembly hall at the same school, so they could go then to start their school day at the regular time (i.e., 9:00 a.m.). According to the user manuals, the three activity wristbands (Fitbit Ace 2, Garmin Vivofit Jr 2, and Xiaomi Mi Band 5) were adjusted on the schoolchildren's wrist of the non-dominant hand. In order to avoid the relative position of the activity wristbands on the wrist influencing the outcomes, they were adjusted in random order varying across schoolchildren (47). Moreover, an accelerometer (ActiGraph wGT3X-BT) was adjusted on the schoolchildren's right hip using an elastic waistband. Activity wristbands/accelerometers were adjusted so they could not move, but overtightening was avoided. On the other hand, in order to avoid potential biases due to schoolchildren's reactivity, the activity wristbands' displays were blinded to hide PA feedback (note that the ActiGraph accelerometer does not have any display). Furthermore, during the waking time, participants were urged to maintain their habitual PA levels, and they were asked to take them off only when they took a bath/shower. Schoolchildren were instructed to wear the activity wristbands/accelerometers for the whole day until bedtime. Schoolchildren were also instructed to remove the activity wristbands/accelerometers and leave them in a plastic box inside their schoolbags just before going to bed. Apart from the verbal instructions, schoolchildren were provided with written instructions together with a diary to record the time they put on and took off the devices throughout the day. In the morning of the following day, the activity wristbands/accelerometers were collected and adjusted onto the next 5–6 schoolchildren following the same protocol.

### 2.4. Statistical analysis

Descriptive statistics for all the variables of the included participants were calculated. Firstly, all the statistical tests assumptions were checked and met (e.g., histograms and Q-Q plots for normality). Furthermore, univariate (i.e., *z* ± 3.0) and multivariate outliers (i.e., Mahalanobis distance) were removed. Afterward, the agreement between the PA scores (i.e., continuous variables) assessed by the activity wristbands (index test) and the accelerometers (reference standard) were calculated as follows: (a) Equivalence test with the 90% confidence interval (CI) method (48); (b) Limits of Agreement (LOA) with its 95% CI (49); (c) Mean Absolute Error (MAE) (50); (d) Mean Absolute Percentage Error (MAPE) (20); and (e) Intraclass Correlation Coefficient (ICC), and its 95% CI, by a two-way random effects model with absolute agreement and single measurement [also known as ICC (1, 2)] (51). Additionally, LOA plots, which are the individual participant differences between the two scores plotted against the respective individual means, were performed (52). Heteroscedasticity was also examined objectively by calculating the Pearson's correlation coefficient (*r*) between the absolute differences and the individual means (53). Based on Cohen's (54) benchmarks, a correlation coefficient >0.50 was considered as indicative of heteroscedasticity. Finally, the agreement between the PA scores dichotomized as meeting or not meeting the daily PA recommendations (i.e., 10,000 steps, 12,000 steps, and 60 min of MVPA) (i.e., categorical variables) assessed by the activity wristbands and the accelerometers were calculated as the proportion of agreement (*P*) and kappa coefficient (*k*) (55). Agreement values were interpreted as follows: Equivalence test, when the mean reference standard score is within ± 15% of the mean activity wristband score is considered acceptable (48); MAPE, >15.0% poor, 10.1–15.0% acceptable, 5.1–10.0% good, and 0.0–5.0% excellent (20); ICC, 0.00–0.49 unacceptable, 0.50–0.59 poor, 0.60–0.69 questionable, 0.70–0.79 acceptable, 0.80–0.89 good, and 0.90–1.00 excellent (33); *k*, 0.00–0.39 poor, 0.40–0.59 acceptable, 0.60–0.74 good, and 0.75–1.00 excellent (36). Based on statistical inference, each ICC value was interpreted according to its 95% IC, that means, there was 95% chance that the true ICC value landed on any point between the 95% IC range (51). All statistical analyses were performed using the SPSS version 25.0 for Windows (IBM^®^ SPSS^®^ Statistics), except for the equivalence test where the Jamovi version 2.3 (The Jamovi project, https://www.jamovi.org) was used. The statistical significance level was set at *p* < 0.05.

## 3. Results

### 3.1. General characteristics

**Figure 1:**
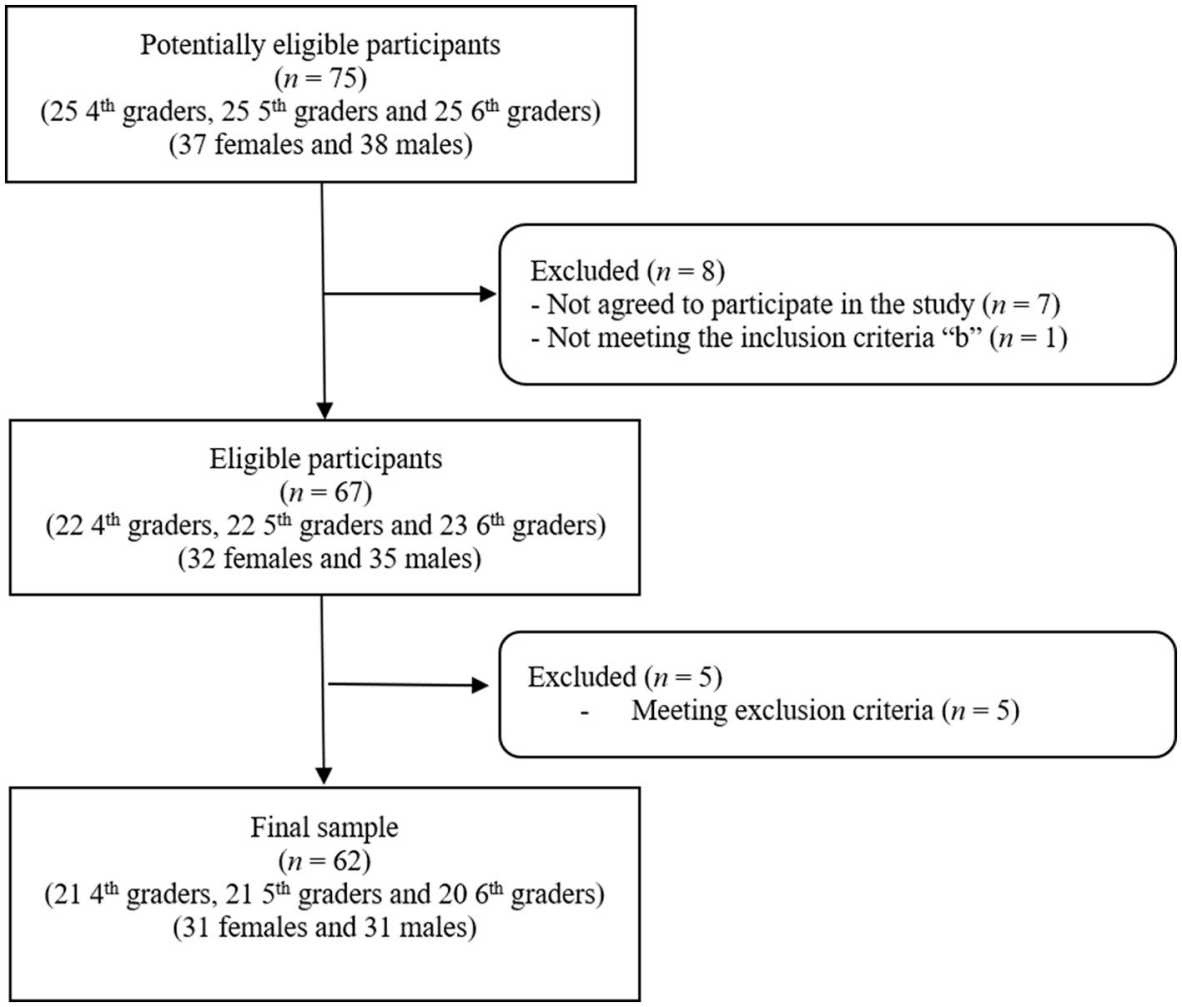
Flow chart of participants included in the present study.

Figure 1 shows the flow diagram of the participants throughout the study. From the 75 schoolchildren that were invited to participate in the present study, 67 schoolchildren agreed and met the inclusion criteria. Since some schoolchildren met at least one exclusion criterion, the final sample consisted of 62 participants (i.e., non-compliance rate of 7.5%). Table 1 shows the general characteristics of the included participants.

**Table 1:** General characteristics of the participants.

	**Eligible sample (*n* = 67)**	**Final sample (*n* = 62)**
Age (years)^a^	10.4 (1.0)	10.4 (1.0)
Grade (4^th^/5^th^/6^th^)^b^	32.8/32.8/34.3	33.9/33.9/32.2
Gender (males/females)^b^	52.2/47.8	50.0/50.0
Body mass (kg)^a^	44.6 (11.0)	44.1 (10.9)
Body height (cm)^a^	145.1 (8.5)	144.8 (8.7)
Body mass index (kg/m^2^)^a^	21.0 (3.8)	20.8 (3.8)
Overweight/obesity (no/yes)^b^	56.7/43.3	58.1/41.9
Non-dominant hand (left/right)^b^	88.1/11.9	87.1/12.9
Daily steps (counts)^a, c^	9,177.3 (2,772.0)	8,948.7 (2,563.5)
Meeting ≥ 10,000 steps/day (yes/no)^b, c^	32.8/67.2	30.6/69.4
Meeting ≥ 12,000 steps/day (yes/no)^b, c^	14.9/85.1	11.3/88.7
Daily MVPA (min)^a, c^	54.3 (22.5)	52.6 (21.0)
Meeting ≥ 60 min of MVPA (yes/no)^b, c^	37.3/62.7	35.5/64.5

Data are reported as mean (standard deviation)^a^ or percentage^b^. MVPA, Moderate-to-vigorous physical activity. ^c^Data recorded by the accelerometer ActiGraph wGT3X-BT.

### 3.2. Validity of the activity wristbands for estimating daily physical activity

**Figure 2:**
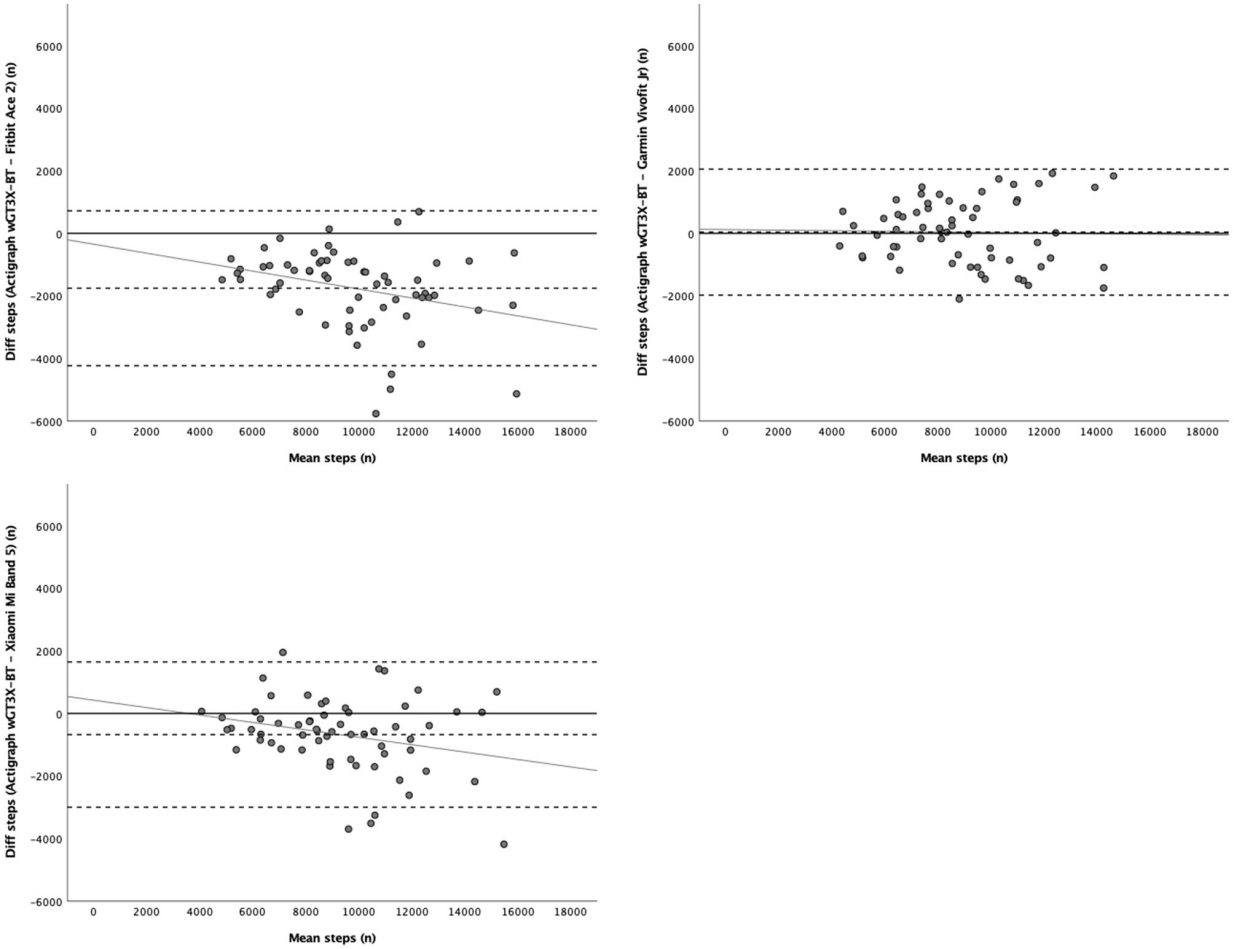
Limits of agreement plots of the activity wristbands for estimating daily steps. The middle-dashed line indicates the mean difference (systematic bias) between step scores assessed by the three activity wristbands and the ActiGraph wGT3X-BT (reference standard) and the upper and lower dashed lines indicate the limits of agreement (95% confidence interval).

**Figure 3:**
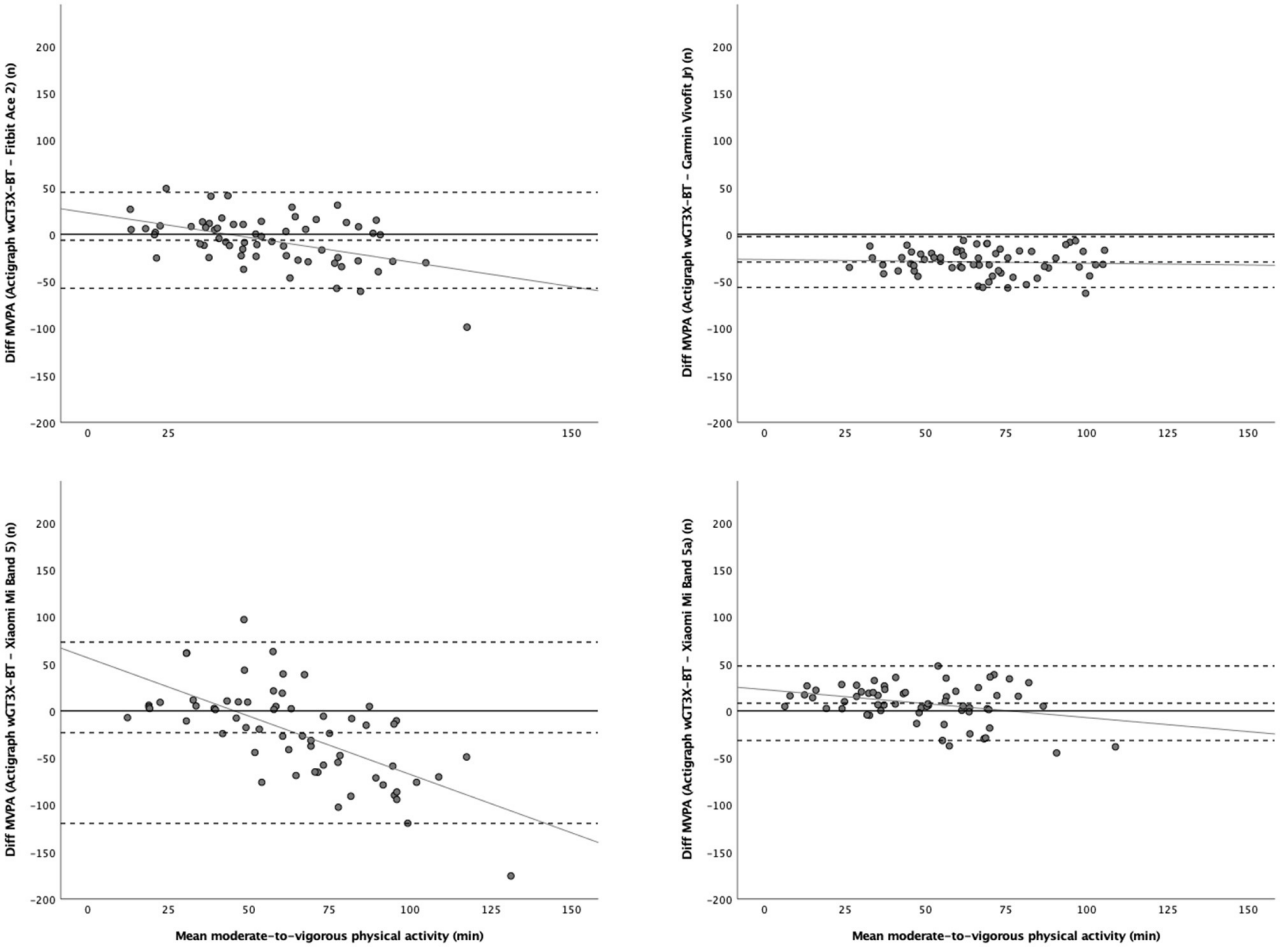
Limits of agreement plots of the activity wristbands for estimating daily moderate-to-vigorous physical activity. The middle-dashed line indicates the mean difference (systematic bias) between moderate-to-vigorous physical activity (down-right plot for brisk walking time) scores assessed by the three activity wristbands and the ActiGraph wGT3X-BT (reference standard) and the upper and lower dashed lines indicate the limits of agreement (95% confidence interval).

Table 2 shows the validity of the activity wristbands for estimating daily PA in primary schoolchildren under free-living conditions. The results showed that the validity of the daily step scores estimated by the Garmin Vivofit Jr 2 and Xiaomi Mi Band 5 were good and acceptable (e.g., scores inside the 90% CI of the equivalence test, MAPE = 9.6/11.3%, and 95% IC of the ICC = 0.87/0.73), respectively. However, the results showed that the validity of the daily step scores estimated by the Fitbit Ace 2 were poor/unacceptable (e.g., scores outside the 90% CI of the equivalence test, MAPE = 21.1%, and 95% IC of the ICC = 0.00). Furthermore, the results showed that the validity of the daily MVPA scores estimated by the three activity wristbands were poor/unacceptable (e.g., scores outside the 90% CI of the equivalence test, MAPE = 36.6–90.3%, and 95% IC of the ICC = 0.00–0.41). Figures 2, 3 show the LOA plots for the daily steps and MVPA, respectively. Pearson's correlation coefficients did not show heteroscedasticity (*r* = 0.06–0.50), except in MVPA with the Xiaomi Mi Band 5 (*r* = 0.59; Supplementary material 1).

**Table 2:** Validity of the activity wristbands for estimating daily physical activity (*n* = 62).

**Instrument**	**Mean (SD)**	**Equivalence test (90% IC)**	**LOA (95% CI)**	**MAE**	**MAPE**	**ICC (95% CI)**
***Steps (n)***						
ActiGraph wGT3X–BT	8,948.7 (2,563.5)	−1,342.31, 1,342.31	–	–	–	–
Fitbit Ace 2	10,703.8 (2,938.1)	−2,023.00, −1,487.00	−1,755.2 (−4,231.5, 721.1)	1,793.6	21.1	0.75 (0.00, 0.92)
Garmin Vivofit Jr 2	8,910.1 (2,586.1)	−179.00, 256.00	38,6 (−1,974.7, 2,051.9)	862.4	9.6	0.92 (0.87, 0.95)
Xiaomi Mi Band 5	9,626.1 (2,871.1)	−929.00, −426.00	−677,5 (−2,999,7, 1,644,7)	993.5	11.3	0.88 (0.73, 0.94)
***Moderate-to-vigorous physical activity (min)***						
ActiGraph WGT3X-BT	52.6 (21.0)	−7.89, 7.89	–	–	–	–
Fitbit Ace 2	59.0 (32.1)	−11.87, −0.85	−6,4 (−57.4, 44.6)	20.0	43.7	0.53 (0.33, 0.69)
Garmin Vivofit Jr 2	82.1 (21.7)	−32.39, −26.53	−29,5 (−56.5, −2.5)	29.5	73.7	0.41 (0.00, 0.75)
Xiaomi Mi Band 5	75.9 (46.3)	−33.70, −12.83	−23,3 (−119.7, 73.1)	40.3	90.3	0.05 (0.00, 0.27)
Xiaomi Mi Band 5^a^	44.5 (26.9)	3.86, 12.42	8,1 (−31.5, 47.7)	17.6	36.6	0.62 (0.41, 0.76)

SD, Standard deviation; LOA, Limits of agreement; 90/95% CI, 90/95% confidence interval; MAE, Mean absolute error; MAPE, Mean absolute percentage error; ICC, Intraclass correlation coefficient;

a Brisk walking time (min).

Table 3 shows the validity of the activity wristbands for estimating the daily PA recommendations in primary schoolchildren under free-living conditions. The results to correctly classify schoolchildren as meeting or not meeting the daily 10,000/12,000-step-based recommendations showed that the validity of the daily step scores estimated by the Garmin Vivofit Jr 2 and Xiaomi Mi Band 5 wristbands were excellent/good and good/acceptable (e.g., Garmin Vivofit Jr 2*, k* = 0.75/0.62; Xiaomi Mi Band 5, *k* = 0.73/0.53), respectively. However, for the Fitbit Ace 2 the results were acceptable/poor (e.g., 10,000 steps, *k* = 0.48; 12,000 steps, *k* = 0.36). Furthermore, regarding the daily MVPA-based recommendation, the results showed that the validity of the MVPA scores estimated by the three activity wristbands were poor-acceptable (e.g., Fitbit Ace 2, *k* = 0.54; Garmin Vivofit Jr 2, *k* = 0.17; Xiaomi Mi Band 5-MVPA score/brisk walking score, *k* = −0.03/0.41).

**Table 3:** Validity of the activity wristbands for estimating daily physical activity recommendations (*n* = 62).

		**ActiGraph wGT3X-BT**										
		**10,000 steps**				**12,000 steps**				**60 min of MVPA**		
**Instrument**		**%TP**	***P***	***k***		**%TP**	***P***	***k***		**%TP**	***P***	***k***
Fitbit Ace 2	10,000 steps	58.1	0.73	0.48^†^	12,000 steps	30.7	0.77	0.36^‡^	60 min of MVPA	45.2	0.77	0.54^†^
Garmin Vivofit Jr 2		35.5	0.89	0.75^†^		12.9	0.92	0.62^†^		85.5	0.50	0.17^*^
Xiaomi Mi Band 5		43.6	0.87	0.73^†^		21.0	0.87	0.53^†^		56.5	0.47	−0.03
Xiaomi Mi Band 5^a^		-	-	-		-	-	-		29.0	0.74	0.41^‡^

MVPA, Moderate-to-vigorous physical activity; %TP, Percentage of total positive cases according to the specified daily activity wristband-based physical activity recommendation; *P*, Proportion of agreement; *k*, Kappa coefficient.

a Brisk walking time (min).

* *p* < 0.05,

‡ *p* < 0.01 and

† *p* < 0.001.

### 3.3. Comparability of the activity wristbands for estimating daily physical activity

Table 4 shows the comparability of the activity wristbands for estimating daily PA in primary schoolchildren under free-living conditions. The results showed that the comparability of the daily step scores estimated by the Garmin Vivofit Jr 2 and Xiaomi Mi Band 5 were acceptable/excellent (e.g., scores inside the 90% CI of the equivalence test, MAPE = 0.1%, and 95% IC of the ICC = 0.70). However, the results showed that the daily step scores of the Fitbit Ace 2 were not comparable with those estimated by the Garmin Vivofit Jr 2 nor Xiaomi Mi Band 5 (e.g., scores of the 95% IC of the ICC = 0.00/0.13). Furthermore, as regards the comparability of the MVPA scores, the results showed that none of the activity wristbands scores were comparable (e.g., scores outside the 90% CI of the equivalence test and 95% IC of the ICC = 0.00–0.32). Pearson's correlation coefficients did not show heteroscedasticity (*r* = 0.09–0.50), except with the MVPA time and brisk walking time estimated by the Xiaomi Mi Band 5 (*r* = 0.60; Supplementary material 1).

**Table 4:** Comparability of the activity wristbands for estimating daily physical activity (*n* = 62).

**Instrument**	**Equivalence test (90% IC)**	**LOA (95% CI)**	**MAE**	**MAPE**	**ICC (95% CI)**
*Steps (n)*	−1,342.31, 1,342.31				
Fitbit Ace 2 - Garmin Vivofit Jr 2	1,557.00, 2,031.00	1,793.8 (−398.3, 3,985.9)	1,800.4	0.2	0.76 (0.00, 0.93)
Fitbit Ace 2 - Xiaomi Mi Band 5	926, 1,230	1,077.7 (−326.4, 2481.8)	1,077.7	0.1	0.91 (0.13, 0.97)
Garmin Vivofit Jr 2 - Xiaomi Mi Band 5	−929, −504	−716.1 (−2,679.8, 1,247.6)	977.7	0.1	0.90 (0.70, 0.96)
*Moderate-to-vigorous physical activity (min)*	−7.89, 7.89				
Fitbit Ace 2 - Garmin Vivofit Jr 2	−27.92, −18.28	−23.1 (−67.6, 21.4)	27.5	0.5	0.49 (0.00, 0.74)
Fitbit Ace 2 - Xiaomi Mi Band 5	−29.89, −3.92	−16.9 (−136.9, 103.1)	50.6	0.7	0.00 (0.00, 0.07)
Fitbit Ace 2 - Xiaomi Mi Band 5^a^	10.41, 18.59	14.5 (−23.3, 52.3)	19.5	0.5^b^	0.71 (0.32, 0.86)
Garmin Vivofit Jr 2 - Xiaomi Mi Band 5	−4.52, 16.91	6.2 (−92.8, 105.2)	40.3	0.6	0.03 (0.00, 0.27)
Garmin Vivofit Jr 2 - Xiaomi Mi Band 5^a^	33.66, 41.54	37.6 (1.1, 74.1)	38.8	0.7	0.33 (0.00, 0.68)
Xiaomi Mi Band 5 - Xiaomi Mi Band 5^a^	19.82, 42.98	31.4 (−75.6, 138.4)	50.2	0.8	0.00 (0.00, 0.17)

LOA, Limits of agreement; 90/95% CI, 90/95% confidence interval; MAE, Mean absolute error; MAPE, Mean absolute percentage error; ICC, Intraclass correlation coefficient;

a Brisk walking time (min).

b Due to zero value in the denominator in a case, the sample size was 61.

Table 5 shows the comparability of the activity wristbands for estimating the daily PA recommendations in primary schoolchildren under free-living conditions. The results to correctly classify schoolchildren as meeting or not meeting the daily 10,000/12,000-step-based recommendations showed that the comparability of the daily step scores estimated by the Fitbit Ace 2/Garmin Vivofit Jr 2 and Xiaomi Mi Band 5 were good/excellent (e.g., 10,000 steps, *k* = 0.72; 12,000 steps, *k* = 0.75) and excellent/good (e.g., 10,000 steps, *k* = 0.77; 12,000 steps, *k* = 0.60), respectively; and for the Fitbit Ace 2 and Garmin Vivofit Jr 2 were acceptable (e.g., 10,000 steps, *k* = 0.57; 12,000 step, *k* = 0.50). However, regarding the daily MVPA-based recommendation, the results showed that none of the activity wristbands scores were comparable (e.g., *k* = −0.19–0.25), except with the Fitbit Ace 2 with the Xiaomi Mi Band 5 (brisk walking time) which were acceptable (e.g., *k* = 0.53).

**Table 5:** Comparability of the activity wristbands for estimating daily physical activity recommendations (*n* = 62).

		**10,000 steps**	
**Instrument**		***P***	***k***
Fitbit Ace 2 - Garmin Vivofit Jr 2	10,000 steps	0.77	0.57^†^
Fitbit Ace 2 - Xiaomi Mi Band 5		0.85	0.72^†^
Garmin Vivofit Jr 2 - Xiaomi Mi Band 5		0.89	0.77^†^
		**12,000 steps**	
		***P***	***k***
Fitbit Ace 2 - Garmin Vivofit Jr 2	12,000 steps	0.82	0.50^†^
Fitbit Ace 2 - Xiaomi Mi Band 5		0.90	0.75^†^
Garmin Vivofit Jr 2 - Xiaomi Mi Band 5		0.89	0.60^†^
		**60 min of MVPA**	
		***P***	***k***
Fitbit Ace 2 - Garmin Vivofit Jr 2	60 min of MVPA	0.60	0.25^‡^
Fitbit Ace 2 - Xiaomi Mi Band 5		0.44	−0.12
Fitbit Ace 2 - Xiaomi Mi Band 5^a^		0.77	0.53^†^
Garmin Vivofit Jr 2 - Xiaomi Mi Band 5		0.58	0.08
Garmin Vivofit Jr 2 - Xiaomi Mi Band 5^a^		0.44	0.13^*^
Xiaomi Mi Band 5 - Xiaomi Mi Band 5^a^		0.37	−0.19

MVPA = Moderate-to-vigorous physical activity; *P*, Proportion of agreement; *k*, Kappa coefficient.

a Brisk walking time (min).

* *p* < 0.05,

‡ *p* < 0.01 and

† *p* < 0.001.

## 4. Discussion

### 4.1. Validity of the activity wristbands for estimating daily physical activity

The results of the present study showed that the validity of the schoolchildren's daily steps estimated by the Garmin Vivofit Jr 2 and Xiaomi Mi Band 5 was good and acceptable, respectively. On the contrary, the validity for the Fitbit Ace 2 estimating schoolchildren's daily steps was poor. Furthermore, the results of the present study showed that the validity of the schoolchildren's daily MVPA estimated by the three activity wristbands was poor/unacceptable. Although the use of activity wristbands to monitor and promote schoolchildren's PA is increasingly widespread, their validity has not been sufficiently studied, especially among primary schoolchildren (20, 56, 57).

Previous studies about the validity of activity wristbands for estimating primary schoolchildren's daily steps and MVPA under free-living conditions showed similar outcomes to the present study. To our knowledge, the study of Schmidt et al. (27) is the only other study that reviewed the validity of a Fitbit wristband (Flex 2; non-dominant wrist) in primary schoolchildren (mean = 8.1, 6–11 years), for which they used the ActiGraph GT9X accelerometer as the reference standard (right hip; Evenson's MVPA threshold). Similar to the results of the present study with the Fitbit Ace 2, Schmidt et al. (27) observed that the Fitbit Flex 2 had a poor validity for estimating both daily steps (e.g., scores were outside the 90% CI of the equivalence test; MAPE = 45.1%; systematic bias = −3,101.3) and MVPA (e.g., scores were outside the 90% CI of the equivalence test; MAPE = 59.9%; systematic bias = −5.2 min). Since data reported in Schmidt et al. (27) was in number of steps per hour and minutes of MVPA per hour, in order to make comparisons, note that their above-mentioned LOA outcomes were adjusted to the valid wear time record of the present study (i.e., 814.7 min).

As far as we know, the study of Yang et al. (26) is the only one that examined the validity of a Xiaomi wristband (Mi Band, but specific model not reported; non-dominant wrist) in primary schoolchildren (mean = 13.0, 10–17 years), for which they used the ActiGraph GT3X-BT accelerometer as the reference standard (right hip; Vanhelst's MVPA threshold). Similar to the results of the present study with the Xiaomi Mi Band 5, while Yang et al. (26) found a relatively low systematic bias for daily steps (i.e., 633.5), it was high for the MVPA (i.e., −42.6). As regards the Garmin wristband, however, to our knowledge, there is no previous study examining the validity of that brand for estimating daily steps or MVPA in primary schoolchildren under free-living conditions. Finally, regarding the validity of other activity wristbands among primary schoolchildren, as far as we know, only Sirard et al. (28) examined the validity of the Movband 2 (dominant wrist) for estimating daily steps in 6-to-12-year-old schoolchildren (mean = 8.6 years) using the ActiGraph GT3X+ accelerometer as the reference standard (right hip). These authors found that the Movband 2 considerably overestimated the primary school children's daily steps (i.e., 2,190.0 steps).

Although the validity results depend on the population and conditions and, thus, should not be generalized, due to the low number of previous studies on the validity of activity wristbands to estimate primary school children's daily steps and MVPA under free-living conditions, the results of the present study have also been compared with available literature with young people (under 18 years) and under structured conditions. To our knowledge, only three previous studies examined the validity of activity wristbands for estimating daily steps and/or MVPA in secondary students (14, 58) and preschool children (59) under free-living conditions. Similar to the results of the present study with the Garmin and Xiaomi activity wristbands, previous studies also found that while the Garmin Vivofit 1 and 3 (58) and the Xiaomi Mi Band 5 (14) had an acceptable validity for estimating daily steps in secondary students (e.g., scores inside the 90% CI of the equivalence test; MAPE = 11.8, 11.5, and 11.4% for Garmin Vivofit 1/3 and Xiaomi Mi Band 5, respectively), it was poor for MVPA (e.g., scores were outside the 90% CI of the equivalence test; MAPE = 22.6%) (14). Moreover, similar to the results of the present study with the Fitbit activity wristbands, Byun et al. (59) also observed that the Fitbit Flex had a poor validity for estimating daily MVPA (e.g., scores were outside the 90% CI of the equivalence test; MAPE = 55.7%) in preschool children.

Regarding previous studies examining the validity of activity wristbands in primary schoolchildren under structured conditions, to our knowledge, only two previous studies were carried out for steps (25, 60) and one for MVPA (29). Contrary to the results of the present study, previous studies found that the activity wristbands Fitbit Charge HR (25), Fitbit Ace, and Moki (60) had good-excellent validity for estimating steps (e.g., mean MAPE = 9.9, 6.0, and 3.6%, respectively). Additionally, Kang et al. (29) found that the Fitbit Charge HR has just acceptable validity for estimating MVPA (e.g., *k* = 0.40). However, these apparent inconsistences between the findings of the present study and those in structured conditions are plausible. While in the studies carried out in controlled conditions individuals were constrained to predefined activities with stable gait patterns (25, 60), or at least most of them, the present study was carried out under a greater variability of motor patterns including a wide range of children's daily life behaviors. Consequently, it is to be expected that the mean error is lower in the first above mentioned case compared with the error in measurement in the second case (20). In this line, systematic reviews have shown that activity wristbands tend to have a higher validity for estimating steps and MVPA under controlled conditions than under free-living conditions (56, 57). However, studies focused solely on controlled conditions may fail in the ecological validation of activity wristbands under free-living conditions (20). Similarly, although nowadays video-based counting and oxygen uptake measured by a portable indirect calorimetry system are considered the “reference standard” for assessing steps and MVPA, respectively (20, 21), these methods are not feasible under free-living conditions (21). Among the large number of methods for the assessment of daily steps and MVPA, today research-grade accelerometers, especially ActiGraph devices, are considered as the most appropriate alternative in free-living conditions (21–24). Consequently, because the main goal of activity wristbands is to monitor and promote children's daily habitual PA, the findings obtained from free-living conditions are closer to reality and, thus, they are more meaningful and useful (61).

The results of the present study to correctly classify schoolchildren as meeting or not meeting the daily 10,000/12,000-step-based recommendations showed that the validity of the Garmin Vivofit Jr 2 and Xiaomi Mi Band 5 activity wristbands were excellent/good and good/acceptable, respectively. However, for the Fitbit Ace 2 the results were acceptable/poor. Furthermore, regarding the daily MVPA-based recommendation, the results showed that the validity of the MVPA scores estimated by the three activity wristbands were poor-acceptable. To our knowledge, previously only Viciana et al. (14) have examined the validity of an activity wristband (i.e., Xiaomi Mi Band 5) to correctly classify individuals (secondary students) as meeting or not meeting the daily PA recommendations (10,000 steps per day and 60 min of MVPA). Similar to the results of the present study with the Xiaomi Mi Band 5, the above-mentioned study found that this activity wristband has an excellent validity for correctly classifying secondary students as meeting or not meeting the daily 10,000-step-based recommendations (e.g., *k* = 0.85). Moreover, Viciana et al. (14) also found that for the MVPA-based recommendation the validity was considerably lower like in the present study (e.g., MVPA/brisk walking outputs: *k* = −0.03/0.17).

Since the MVPA-based guidelines are not easily understood by both schoolchildren and their parents (5), these guidelines have also been translated to simple and easier-to-understand daily step-based recommendations. Particularly among primary schoolchildren, previous studies have found a high accuracy in the translation of the MVPA-based guidelines to about 10,000 (6) or 12,000 steps per day (7). Moreover, as shown in the results of the present study, because activity wristbands tend to have a much lower validity for estimating school children's MVPA than for steps (see discussion above), in order to correctly classify schoolchildren as meeting or not meeting the MVPA-based recommendations, activity wristband-based steps have shown to be considerably more valid than even with the activity wristband MVPA output (13). In this line, for instance, Casado-Robles et al. (11) in a systematic review about consumer-wearable activity tracker-based programs found that most of the studies with a goal-setting strategy set only a step-based goal (81%). Therefore, although activity wristbands present poor validity for estimating MVPA outputs, the results with the Garmin Vivofit Jr 2 and Xiaomi Mi Band 5 are promising for public health policies, in order to set daily step-based targets and receive accurate feedback on their achievement among primary schoolchildren. Specifically, they allow for knowing if primary schoolchildren are achieving the PA recommendation and, therefore, its consequent health benefits (1).

### 4.2. Comparability of the activity wristbands for estimating daily physical activity

The results of the present study showed that the comparability of the daily step scores estimated by the Garmin Vivofit Jr 2 and Xiaomi Mi Band 5 were adequate. On the contrary, the daily step scores of the Fitbit Ace 2 were not comparable with those estimated by the Garmin Vivofit Jr 2 and Xiaomi Mi Band 5. However, with the aim of simply classifying schoolchildren as meeting or not meeting the daily step-based recommendations, the results showed that the three activity wristbands scores were comparable. As regards the comparability of the MVPA scores, however, the results showed that none of the activity wristbands scores were comparable based on both continuous and dichotomous (60-min of MVPA recommendation) variables (exceptionally with the Fitbit Ace 2 and Xiaomi Mi Band 5 -brisk walking time- that were just acceptable).

Although the use of different activity wristbands to monitor and promote school children's PA is commonly used in contexts with economic constrains such as in physical education where each student uses his/her own device (14), to our knowledge, unfortunately, there are no previous topic-related studies in primary schoolchildren. As far as we know, the study of Viciana et al. (14) is the only that examined the comparability of an activity wristband (Xiaomi Mi Band 5), but it was compared with smartwatches, as well as in a sample of secondary students. Similar to the results of the present study, for example, while for the daily steps the Xiaomi Mi Band 5 and Samsung Galaxy Watch Active 2 were comparable [e.g., continuous variable: MAPE = 8.4; ICC = 0.98 (0.91–0.99); LOA = −397.8 (−1,525.2, 729.6); dichotomous 10,000-step recommendation: e.g., *k* = 0.85], for the MVPA score was not [e.g., continuous: MAPE = 86.0; ICC = 0.10 (0.00, 0.31); LOA = 29.4 (−114.7, 55.9); dichotomous 60-min recommendation: *k* = 0.04/0.17].

Therefore, considering that the Garmin Vivofit Jr 2 and Xiaomi Mi Band 5 were comparable for estimating daily steps, apart from the price, technical characteristics, and options offered by the different activity wristbands, this could also be an important reason to select one or another for a particular aim (14). For instance, battery duration, attractive screen, goal settings, reminders, or the data registered in the application, among others, could be essential to consider (11). Moreover, in settings such as in physical education where the only economical possible way is that each student uses his/her own device (i.e., already purchased), for instance, the Garmin Vivofit Jr 2 and Xiaomi Mi Band 5 could be used interchangeably to monitor and promote daily steps among primary schoolchildren.

### 4.3. Strengths and limitations

An important strength of the present study was being, to our knowledge, the first one to examine the validity of primary school children's daily steps and MVPA scores estimated by the activity wristbands specifically designed for this population (i.e., Fitbit Ace 2 and Garmin Vivofit Jr 2) under free-living conditions. Moreover, as far as we know, it is also the first study to examine the validity of activity wristbands scores for classifying primary school children as meeting or not meeting the PA recommendations, which is a very relevant issue for those responsible for PA promotion programs in order to evaluate and set targets (11). Finally, to the best of our knowledge, the present study is the first one to examine the comparability of the activity wristband scores among primary schoolchildren, which is another important issue because for feasible reasons are commonly used in contexts such us in physical education or large-scale research studies with different activity wristbands (30, 31). Therefore, the present study allows for addressing important gaps in the scientific literature to date.

However, the present study has some limitations. Firstly, a non-probability and relatively small sample has been used, which limits the generalizability of the obtained outcomes to the particular studied setting (i.e., primary schoolchildren with similar characteristics and PA patterns). However, due to the human and material resource restrictions, a probability and larger sample could not be examined. Secondly, another common limitation in this kind of studies is related to heteroscedasticity, that is, the measurement error related to the magnitude of the measured variables (53). Normally participants who score the highest PA values show the greatest amount of measurement error (in the units of measurement) (53). Therefore, since activity wristbands are designed to promote users' PA, schoolchildren could have reacted doing some more PA during the monitoring period and, potentially, introducing bias in the outcomes of the present study. However, in the present study the activity wristbands' displays were blinded to hide PA feedback (note that the ActiGraph accelerometer does not have any display), as well as participants were urged to maintain their habitual PA levels. Moreover, when heteroscedasticity was examined objectively, the results showed that it was not the issue in the present study, except in only 2 out of 16 examined variables that had a moderate heteroscedasticity.

Finally, although ActiGraph accelerometers have been highlighted as the most common and valid method for objectively assessing schoolchildren' PA levels under free-living conditions (24, 46), today there is no strong consensus about numerous methodological data collection and processing criteria (24), which have shown to considerably affect the PA scores (62, 63). Regarding data collection criteria, for instance, the adopted accelerometer placement in the present study (i.e., right hip) might have affected school children's PA scores and, consequently, the validity outcomes, especially considering that activity wristbands were placed in a different part of the body (i.e., on the non-dominant wrist). In a systematic review about the topic, Migueles et al. (63) found that ActiGraph accelerometer-based PA cut-points showed a higher validity when devices were placed on the school children's hips compared to the wrist. Therefore, since in the present study ActiGraph accelerometer-measured PA scores were used as a reference standard, the device placement that has shown the best validity (i.e., hip) was chosen. As regards the processing criteria, for example, the *epoch* length has demonstrated to affect schoolchildren's daily MVPA levels, showing long *epochs* (e.g., 60 seconds) to be statistically significantly lower than those with short *epochs* (e.g., ≤15 seconds) (62). Since schoolchildren's PA patterns are characterized by short bursts of quickly changing activity, 1-to-15-second *epochs* have been recommended (24). However, due to the fact that with extremely short *epochs* there is not enough time to characterize the intensity of any movement (62), in the present study the 15-second *epoch* was set. As another example of processing criteria, the MVPA cut-point has also shown to affect drastically the MVPA scores (63). Even though there is still no consensus within the area of knowledge regarding the selection of MET intensity thresholds for schoolchildren (64), the 4-MET threshold has been suggested as being more appropriate to account for their higher level of resting energy expenditure compared with adults (64). Moreover, there is growing evidence that in schoolchildren brisk walking, which is considered a key behavioral marker of moderate PA, is related to an energy cost of approximately 4 METs (64). According to the cross-validation study performed by Trost et al. (44), the Evenson threshold has demonstrated the best evidence supporting score validity for assessing MVPA with short *epochs* and considering 4-METs among schoolchildren. Later, Romanzini et al. (46) in a calibration study with schoolchildren provided more support for continued use of Evenson's thresholds. Therefore, although different ActiGraph accelerometer-based MVPA cut-points are available, in the present study the Evenson's threshold was used because it is based on 4-METs threshold and, also, it has been shown to be the most valid among schoolchildren (44, 46). Therefore, although the best current evidence-based decisions were adopted in the present study (24), the reference standard may contribute to the variability of the studied activity wristbands validity outcomes.

Due to these aforementioned limitations, further studies should be performed to improve the knowledge about the validity of the studied activity wristbands and new models for the recording of PA parameters. Moreover, since other PA outputs such as heart rate, distance, or energy expenditure, are commonly used in the activity wristbands, future studies should also examine the validity of these scores in primary schoolchildren under free-living conditions. Furthermore, in the case of activity wristband companies allowing work with raw data, it would be interesting for future studies by independent researchers to develop and examine more accurate algorithms according to the characteristics of each particular population. Alternatively, these companies would allow at least to set some calibration in their applications (e.g., different cut-points for the intensity-related PA), or even new algorithms could be created based on the reported data (65), so future research studies can develop most accurate data according to the characteristics of each particular user. Finally, although wrist-worn wearables have shown to be most effective in promoting schoolchildren's PA than those placed on the hip (11), on the contrary, at least in part, due to undesired movements of arms, the hip has shown to be a better place of achieving an accurate PA measurement (24). Thus, future research studies could develop and compare the validity of algorithms based on activity wristbands according to the body placement. Then, in order to obtain a better validity of PA scores, wearables could be based on two devices, one placed on the hip (or any place on the body that obtains accurate results) for recording and analyzing the information and a second device on the wrist that, based on the outcomes from the first device, report and interact with the user (i.e., similar to chest strap heart rate monitors).

## 5. Conclusions

The Garmin Vivofit Jr 2 showed a good validity for estimating daily primary schoolchildren's steps, as well as accurately classifying them as meeting or not meeting the step-based recommendations. Alternatively, if there are economic constraints, the Xiaomi Mi Band 5 (i.e., the lowest priced studied model) showed an acceptable validity for estimating both daily steps and step-based recommendations, as well as having comparable step output with the Garming Vivofit Jr 2. However, despite being specially designed for primary schoolchildren, the Fitbit Ace 2 has not shown an acceptable validity for estimating daily steps and step-based recommendations. None of the three activity wristbands examined in the present study showed an adequate validity for estimating daily MVPA, as well as the validity for MVPA-based recommendation tending to be considerably lower than for step-based recommendations. This highlights the potential of the Garmin Vivofit Jr 2 for monitoring primary schoolchildren's daily steps, offering a feasible alternative to the research-grade accelerometers. Furthermore, this activity wristband could be used during PA promotion programs to provide accurate feedback to schoolchildren to ensure their accomplishment with the PA recommendations.

## Data availability statement

The dataset used and analyzed during the current study are available from the corresponding author on reasonable request.

## Ethics statement

The studies involving human participants were reviewed and approved by Ethical Committee for Human Studies at the University of Granada (1252/CEIH/2020). Written informed consent to participate in this study was provided by the participants' legal guardian.

## Author contributions

CC-R was responsible for collecting the data. DM-V carried out the analysis and interpretation of the data and drafted the manuscript. All authors contributed to the conception and design of the study, editing and revising the manuscript, and read and approved the final manuscript.
