# Supplementary material for: Validity of activity wristbands for estimating daily physical activity in primary schoolchildren under free-living conditions: School-Fit study
Source: Front Public Health. 2023 Jul 24;11:1211237. doi: 10.3389/fpubh.2023.1211237 (PMC10405174; doi:10.3389/fpubh.2023.1211237)
Supplement: Supplementary file 1 [file Table_1.docx]

Supplementary Material

Validity of activity wristbands for estimating daily physical activity in primary schoolchildren under free-living conditions: School-Fit study

Daniel Mayorga-Vega, Carolina Casado-Robles, Santiago Guijarro-Romero, Jesús Viciana

*** Correspondence:** Santiago Guijarro-Romero: santigr93@gmail.com

| Supplementary Table 1. Pearsonʼs correlation coefficient (*r*) between the absolute differences and the individual means (*n* = 62) | | |
| --- | --- | --- |
|  | Validity | |
| Instrument | Steps (n) | MVPA (min) |
| Fitbit Ace 2 | 0.34‡ | 0.42‡ |
| Garmin Vivofit Jr 2 | 0.50† | 0.06 |
| Xiaomi Mi Band 5 | 0.35‡ | 0.59†/0.27*^a^ |
|  | Comparability | |
|  | Steps (n) | MVPA (min) |
| Fitbit Ace 2 - Garmin Vivofit Jr 2 | 0.34‡ | -0.29* |
| Fitbit Ace 2 - Xiaomi Mi Band 5 | 0.09 | 0.50† |
| Fitbit Ace 2 - Xiaomi Mi Band 5^a^ | - | 0.40‡ |
| Garmin Vivofit Jr 2 - Xiaomi Mi Band 5 | 0.35‡ | 0.16 |
| Garmin Vivofit Jr 2 - Xiaomi Mi Band 5^a^ | - | -0.24 |
| Xiaomi Mi Band 5 - Xiaomi Mi Band 5^a^ | - | 0.60† |
| *Note*. MVPA = Moderate-to-vigorous physical activity; ^a^ Brisk walking time (min).  * *p* < 0.05, ‡ *p* < 0.01, and † *p* < 0.001 | | |
